# *Candidatus* Neoehrlichia mikurensis in ticks and rodents from urban and natural habitats of South-Western Slovakia

**DOI:** 10.1186/s13071-015-1287-2

**Published:** 2016-01-04

**Authors:** Zuzana Hamšíková Svitálková, Danka Haruštiaková, Lenka Mahríková, Michala Mojšová, Lenka Berthová, Mirko Slovák, Elena Kocianová, Muriel Vayssier-Taussat, Mária Kazimírová

**Affiliations:** Institute of Zoology, Slovak Academy of Sciences, Dúbravská cesta 9, 84506 Bratislava, Slovakia; Institute of Biostatistics and Analyses, Faculty of Medicine and Faculty of Science, Masaryk University, Kamenice 3, 62500 Brno, Czech Republic; Department of Zoology, Faculty of Natural Sciences, Comenius University, Mlynská Dolina B-1, 84215 Bratislava, Slovakia; Institute of Virology, Slovak Academy of Sciences, Dúbravská cesta 9, 84505 Bratislava, Slovakia; UMR BIPAR, Anses, 23 Avenue du Général de Gaulle, 94700 Maisons-Alfort, France

**Keywords:** *Candidatus* Neoehrlichia mikurensis, *Ixodes ricinus*, Rodents, Tick-borne Pathogen

## Abstract

**Background:**

*Candidatus* Neoehrlichia mikurensis (CNM) is an emerging tick-borne pathogen causing severe disease in immunocompromised patients. In Europe, *Ixodes ricinus* is the primary vector and rodents act as reservoir hosts. New data on the prevalence of CNM in ticks and rodents contribute to the knowledge on the distribution of endemic areas and circulation of the bacterium in natural foci.

**Methods:**

Questing ticks were collected and rodents were trapped in urban/suburban and natural habitats in South-Western Slovakia from 2011 to 2014. DNA from questing and rodent-attached ticks and rodent tissues were screened for CNM by real-time PCR. Rodent spleen samples positive for CNM were characterised at the *groEL* gene locus. Spatial and temporal differences in CNM prevalence in ticks and rodents and co-infections of ticks with CNM and *Anaplasma phagocytophilum* were analysed.

**Results:**

The presence of CNM was confirmed in questing and rodent-attached *I. ricinus* ticks and in rodents. Total prevalence in both ticks and rodents was significantly higher in the natural habitat (2.3 % and 10.1 %, respectively) than in the urban/suburban habitat (1.0 % and 3.3 %, respectively). No seasonal pattern in CNM prevalence in ticks was observed, but prevalence in rodents was higher in autumn than in spring. CNM was detected in *Apodemus flavicollis, Myodes glareolus, Microtus arvalis* and *Micromys minutus,* with the highest prevalence in *M. arvalis* (30 %). By screening CNM dissemination in rodent tissues, infection was detected in lungs of all specimens with positive spleens and in blood, kidney, liver and skin of part of those individuals. Infection with CNM was detected in 1.3 % of rodent attached *I. ricinus* ticks. Sequences of a fragment of the *groEL* gene from CNM-positive rodents showed a high degree of identity with sequences of the gene amplified from ticks and infected human blood from Europe. Only 0.1 % of CNM-positive questing ticks carried *A. phagocytophilum.* Ticks infected with CNM prevailed in the natural habitat (67.2 %), whereas ticks infected with *A. phagocytophilum* prevailed in the urban/suburban habitat (75.0 %).

**Conclusion:**

The study confirmed the circulation of CNM between *I. ricinus* ticks and rodents in South-Western Slovakia, and indicates a potential risk of contracting human infections.

## Background

*Candidatus* Neoehrlichia mikurensis (CNM) (Rickettsiales, Anaplasmataceae) is an emerging tick-borne pathogen of medical importance in Eurasia [1–4]. Severe diseases have been reported mainly in immunocompromised human patients [3–6]. In addition to humans, neoehrlichiosis was diagnosed in one dog [7].

CNM was first detected in *Ixodes ricinus* ticks from the Netherlands in the late 1990s. It was originally ranked among *Ehrlichia*-like species and named the ‘Schotti-variant’ [8]. Recently, CNM has been found in questing and host-attached ticks (mainly *Ixodes* spp.) and rodents throughout several European, Asian and African countries [3, 4]. The occurrence of CNM was also confirmed in museum-archived *I. ricinus* collected in Moldova in 1960 [9], suggesting that the bacterium was present in tick populations for a long time before its discovery.

Recent studies from Europe indicate that CNM is a common and widespread tick-borne bacterium occurring in different habitat types within the distribution area of *I. ricinus* and *I. persulcatus* [10–22]. *Ixode*s *ricinus* is considered to be the primary vector of CNM in Central Europe [1, 4, 23]. Rodents play an important role as competent zoonotic reservoirs maintaining the natural life cycle of the bacterium [20, 23–25]. In addition to rodents, CNM-infected ticks have been found infesting hedgehogs, larger mammals (ruminants, wild boar) and birds, that, by carrying infected ticks, can contribute to the geographical spread of the infection [12, 26–28].

In Slovakia, CNM (classified as the “Schotti variant”) was detected first in an *I. ricinus* nymph attached to a song thrush [29]. Since then, detections of the bacterium in *I. ricinus* and rodents have been common [18, 30–32], confirming the existence of endemic CNM foci in Slovakia and suggesting the occurrence of subclinical human infections.

In general, habitat structure and the presence and abundance of reservoir and non-reservoir hosts may play an important role in local variations in the proportion of ticks infected with one or multiple microorganisms [33]. Although the prevalence of CNM in questing *I. ricinus* and rodents in Europe has been found to vary depending on site and habitat*,* the knowledge on the factors driving its geographic distribution and circulation in natural foci is still limited. Furthermore, low rates of co-infections of CNM and *Anaplasma phagocytophilum,* another member of Anaplasmataceae (Rickettsiales), reported for ticks from several European sites [10, 17, 19, 34, 35] suggest that the two bacteria do not share the same reservoir hosts.

The aims of this study were: (1) to determine the prevalence of CNM in questing *I. ricinus* and rodents in urban/suburban and natural habitats in South-Western Slovakia; (2) to determine co-infections of ticks with CNM and *Anaplasma phagocytophilum*; and (3) to assess the role of rodents in the natural circulation of CNM.

## Methods

### Study area, tick sampling and rodent trapping

The study area is situated in the Small Carpathians Mountains (SW Slovakia). Three 100 m^2^ transects (B1, B2, B3) and a 200 m^2^ transect (B5) were selected for tick collection in an urban/suburban habitat in the northern part of the Bratislava town, in the south-western foothills of the Small Carpathians Mountains (48.17–48.20°N, 17.07–17.10°E, altitude 202–334 m a.s.l.). Three 100 m^2^ transects (F1, F2, F3) were set in a non-fragmented deciduous forest (area Fúgelka) located near the village of Dubová, at a distance of about 40 km from Bratislava (48.37–48.38°N, 17.30–17.32°E, altitude 336–436 m a.s.l.). For more details, see [36].

Ticks and rodents were gathered, identified and processed during a study aimed at their screening for multiple pathogens [36]. Briefly, questing tick collections were carried out by dragging a 1 m^2^ sized white wool blanket over the vegetation along transects B1–B3 and F1–F3 from April–June and September–October of 2011–2013. Additional collections were made in transects B1–B3 in July and August 2013. Random tick collections were carried out in transect B5 from April to June 2011.

Rodents were live-trapped by using Swedish bridge metal traps set in lines along the tick collection transects (except B5) in spring and autumn of 2012, 2013 and 2014 (in total, 1,800 trap/nights in the urban/suburban habitat and 1,900 trap/nights in the natural habitat). Rodent handling was described in [36]. Blood was taken from *sinus orbitalis* from anaesthetised rodents and stored in 70 % ethanol. Rodents were examined for ticks, which were stored in 70 % ethanol and identified. Necropsy of euthanized rodents was carried out. The spleen was stored in 70 % ethanol, and lungs, liver, kidney and skin samples from ears were stored frozen at −80 °C.

### DNA extraction and detection of CNM

DNA was isolated from individual ticks and rodent tissues by the Macherey-Nagel NucleoSpin® Tissue kit (Düren, Germany) following the manufacturer’s instructions. Quantity and quality of the extracted DNA samples were determined with a spectrophotometer Nanodrop 2000c. For more details, see [36].

DNA samples from questing and rodent-attached ticks, rodent spleens and skin were screened for the presence of CNM with a real-time polymerase chain reaction (real-time PCR) targeting a 99-bp fragment of the *groEL* gene [12, 13]. From rodent individuals with CNM-positive spleen or ear biopsies, blood, lungs, kidney and liver were also analysed. The PCR reaction was carried out in a volume of 25 μl in the real-time PCR machine CFX96 Real-Time PCR System (Bio-Rad, Hercules, CA, USA) by using the HotStarTaq PCR kit (Qiagen, Hilden, Germany). The following three primers were used: NMikGroEL-F2 5′-CCTTGAAAATATAGCAAGATCAGGTAG-3′, NMikGroEL rev1 5′-CCACCACGTAACTTATTTAGCACTAAAG-3′ and NMikGroEL rev2 5′-CCACCACGTAACTTATTTAGTACTAAAG-3′. The complementary probe was NMikGroEL-P2a 5′-FAM-CCTCTACTAATTATTGCTGAAGATGTAGAAGGTGAAGC-BHQ1-3′. The PCR was set at the following parameters: initial denaturation at 95 °C for 5 min, 40 cycles of a denaturation period at 95 °C for 15 s and a 1 min annealing period at 60 °C. Negative (molecular grade water) and positive (DNA of naturally CNM-infected *I. ricinus* or rodent spleen) were included in each run. Samples were considered positive with an exponential rise of the curve and a ct-value (threshold cycle) <37.5. Selected positive samples were confirmed by conventional PCR amplifying a 1,024-bp long fragment of the *groEL* gene, as previously described [7].

### Sequencing

PCR products were sent for sequencing to GATC Biotech Company (Germany). Sequences were compared with known sequences listed in the GenBank nucleotide sequence databases by using the BLAST search option at the National Center for Biotechnology Information (www.ncbi .nlm.nih.gov/BLAST).

### Statistical analyses

Differences in CNM prevalence in questing ticks and rodents between habitats and transects, between seasons and years, and between rodent species and sexes were analysed by Fisher’s exact test. Only rodents with positive spleens were considered as CNM positive and included in the analyses. *P* < 0.05 was regarded as significant. The 95 % confidence intervals for prevalence in questing ticks and rodents were computed using a bootstrap technique. Logistic regression was used to estimate the effect of habitat type, year, season and tick developmental stage on the probability of tick infection and the effect of habitat type, year, season, rodent species and sex on the probability of rodent infection. Results on the presence of *A. phagocytophilum* in the same questing ticks, available from a previous study [36], were used to calculate co-infections with CNM and analyse the two bacteria dependant on habitat type using Fisher’s exact test. Statistical analyses were performed with IBM SPSS Statistics, version 22 and Statistica software, version 12.

## Results

### CNM prevalence in questing ticks

**Fig. 1:**
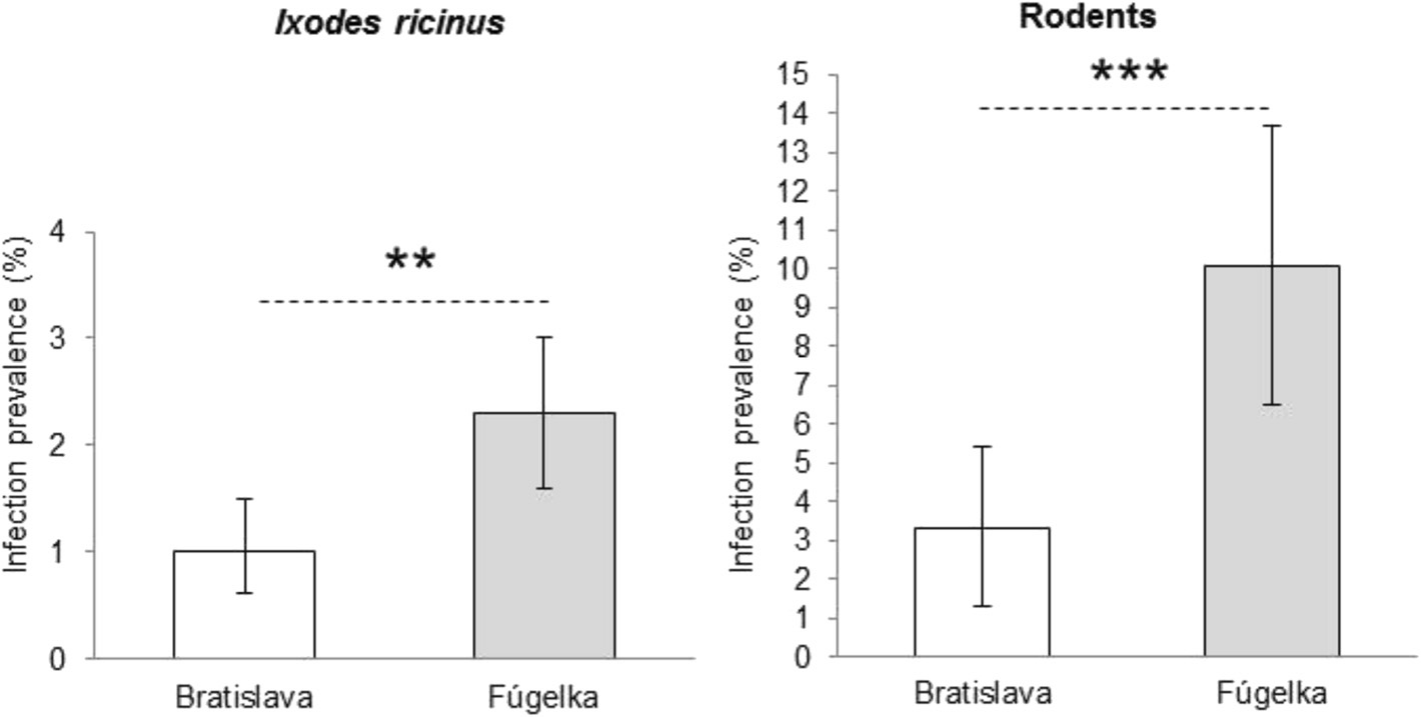
Total prevalence of CNM in questing ticks and rodents in Bratislava and Fúgelka. Legend: Error bars indicate 95 % confidence intervals; ** indicates significant difference between habitats for infection rates in ticks (Fisher’s exact test, *P* = 0.002); *** indicates significant difference between habitats for infection rates in rodents (Fisher’s exact test, *P* = 0.001)

In total, 3,874 and 75 individual *Ixodes ricinus* and *Haemaphysalis concinna* ticks, respectively, were examined for the presence of CNM: 2,034 *I. ricinus* and 47 *H. concinna* from the urban/suburban habitat and 1,840 *I. ricinus* and 28 *H. concinna* from the natural habitat. Only *I. ricinus* were found positive for CNM, with an overall infection rate of 1.6 % (95 % CI: 1.2–2.0 %). The prevalence in the two habitat types differed significantly (Table 1, Fig. 1). Significant difference between the habitats was also found for total prevalence in nymphs (0.8 %, 95 % CI: 0.4–1.4 % in Bratislava; 2.4 %, 95 % CI: 1.6–3.2 % in Fúgelka; Fisher’s exact test, *P* = 0.002), but not for adult ticks (1.4 %, 95 % CI: 0.6–2.4 % in Bratislava; 2.0 %, 95 % CI: 0.9–3.4 % in Fúgelka; Fisher’s exact test, *P* = 0.476). By comparing CNM infection rates in ticks from 3 years (2011–2013), a significant difference was revealed only for the total prevalence at Fúgelka (Table 1).

**Table 1:** Prevalence of CNM in questing *Ixodes ricinus* per site in 2011–2013

		2011		2012		2013		Fisher’s	Total	
Site		% (pos/ex)	95 % CI	% (pos/ex)	95 % CI	% (pos/ex)	95 % CI	exact test	% (pos/ex)	95 % CI
Bratislava	Nymphs	0.9 (6/664)	0.3–1.7	1.0 (2/195)	0.0–2.6	0.7 (3/455)	0.0–1.5	0.845	0.8 (11/1314)	0.4–1.4
	Females	2.0 (4/196)	0.5–4.1	2.0 (1/49)	0.0–6.1	2.0 (2/102)	0.0–4.9	1.000	2.0 (7/347)	0.6–3.5
	Males	1.0 (2/207)	0.0–2.4	1.5 (1/68)	0.0–4.4	0.0 (0/98)		0.587	0.8 (3/373)	0.0–1.9
	Adults total	1.5 (6/403)	0.5–2.7	1.7 (2/117)	0.0–4.3	1.0 (2/200)	0.0–2.5	0.909	1.4 (10/720)	0.6–2.4
	Total	1.1 (12/1067)	0.6–1.8	1.3 (4/312)	0.3–2.6	0.8 (5/655)	0.2–1.5	0.655	1.0 (21/2034)	0.6–1.5
Fúgelka	Nymphs	2.4 (21/867)	1.5–3.5	0.7 (2/270)	0.0–1.9	3.8 (10/263)	1.5–6.5	0.060	2.4 (33/1400)	1.6–3.2
	Females	4.7 (4/85)	1.2–9.4	1.9 (1/52)	0.0–5.8	1.6 (1/61)	0.0–4.9	0.578	3.0 (6/198)	1.0–5.6
	Males	1.0 (1/102)	0.0–2.9	0.0 (0/66)		2.7 (2/74)	0.0–6.8	0.477	1.2 (3/242)	0.0–2.9
	Adults total	2.7 (5/187)	0.5–5.3	0.8 (1/118)	0.0–2.5	2.2 (3/135)	0.0–4.4	0.630	2.0 (9/440)	0.9–3.4
	Total	2.5 (26/1054)	1.6–3.4	0.8 (3/388)	0.0–1.8	3.3 (13/398)	1.8–5.3	0.037	2.3 (42/1840)	1.6–3.0

*(pos/ex)* number of positive/number of examined, *95 % CI* confidence interval

The total CNM infection rate in adult ticks (1.6 %; 95 % CI: 0.9–2.4 %) was similar to nymphs (1.6 %; 95 % CI: 1.2–2.1 %) (Fisher’s exact test, *P* = 1.000). When the sites were analysed separately, the prevalence in adults in Bratislava was higher and in Fúgelka lower than in nymphs (Table 1), but the differences between the stages were not significant (Bratislava: Fisher’s exact test, *P* = 0.256; Fúgelka: Fisher’s exact test, *P* = 0.855).

Total CNM prevalence varied significantly between transects in Bratislava, but not in Fúgelka (Table 2). In Bratislava, the prevalence in transect B1 was significantly lower than in transect B2 (Fisher’s exact test, *P* = 0.003) and B3 (Fisher’s exact test, *P* = 0.004), whereas no difference was found between transects B2 a B3 (Fisher’s exact test, *P* = 0.805).

**Table 2:** Overall prevalence of CNM in *Ixodes ricinus* and rodents per transect in Bratislava and Fúgelka

	% (pos/ex)	95 % CI	% (pos/ex)	95 % CI	% (pos/ex)	95 % CI	Fisher’s exact test
Bratislava/transect^a^	B1		B2		B3		
*I. ricinus*	0.1 (1/766)	0.0–0.4	1.7 (7/404)	0.7–3.0	1.5 (10/658)	0.8–2.4	0.002
Rodents	0.0 (0/2)		2.6 (4/153)	0.7–5.2	4.2 (6/144)	1.4–7.6	0.562
Fúgelka/transect	F1		F2		F3		
*I. ricinus*	3.6 (11/303)	1.7–5.9	2.0 (17/868)	1.2–2.9	2.1 (14/669)	1.0–3.3	0.229
Rodents	10.2 (17/167)	6.0–15.0	9.9 (7/71)	4.2–16.9	10.3 (7/68)	4.4–17.6	1.000

*(pos/ex)* number of positive/number of examined, *95 % CI* confidence interval

^a^only transects B1-B3 where rodent trapping was carried out were included in the analysis

**Fig. 2:**
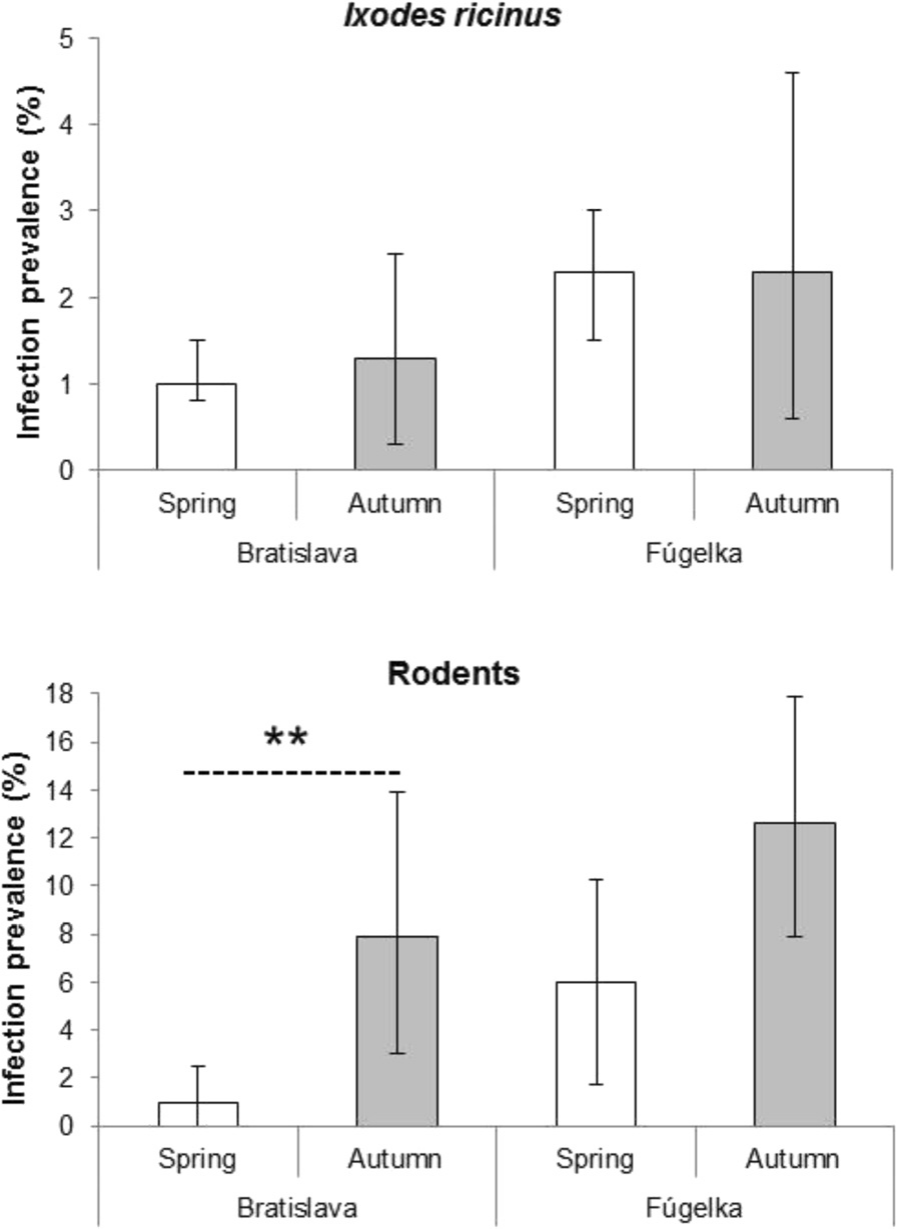
Seasonal differences in prevalence of CNM in questing ticks and rodents in Bratislava and Fúgelka. Legend: Error bars indicate 95 % confidence intervals; ** indicates significant difference between habitats; Bratislava, ticks total (Fisher’s exact test, *P* = 0.556); Fúgelka, ticks total (Fisher’s exact test, *P* = 1.000); Bratislava, rodents total (Fisher’s exact test, *P* = 0.003); Fúgelka, rodents total (Fisher’s exact test, *P* = 0.079)

The analysis of seasonal changes in CNM prevalence did not show any significant differences between ticks collected from April to the beginning of July and those collected from the end of July to October (Fig. 2). Total infection rates in ticks collected from April to the beginning of July significantly differed between the two habitats: 1.0 % (95 % CI: 0.6–1.5 %) in Bratislava; 2.3 % (95 % CI: 1.6–3.1 %) in Fúgelka (Fisher’s exact test, *P* = 0.004). In contrast, the difference in CNM prevalence between habitats was not significant for ticks collected from the end of July to October: 1.3 % (95 % CI: 0.3–2.5 %) in Bratislava; 2.3 % (95 % CI: 0.6–4.6 %) in Fúgelka (Fisher’s exact test, *P* = 0.463).

The analysis of simultaneous effects of habitat, year, season, and tick developmental stage on the probability of infection with CNM by logistic regression resulted in the significant effect of habitat (urban/suburban habitat Bratislava: parameter estimate B = −0.806, exp(B) = 0.447, *P* = 0.003). Variables removed by backward method were season and year.

### Co-infection of *I. ricinus* with CNM and *A. phagocytophilum*

Out of the 3,874 *I. ricinus* ticks considered for analysis of co-infections with CNM and *A. phagocytophilum*, only two females (0.1 %) (one from Bratislava and one from Fúgelka) were infected by both bacteria, 196 ticks (5.1 %) were infected with *A. phagocytophilum* and 61 (1.6 %) with CNM. By comparing the proportions of ticks infected with CNM and *A. phagocytophilum* in the two habitats, significant differences were revealed (Fisher’s exact test, *P* < 0.001). Ticks infected with CNM prevailed in the natural habitat (67.2 %; 41 out of 61), whereas ticks infected with *A. phagocytophilum* prevailed in the urban/suburban habitat (75.0 %; 147 out of 196).

### CNM prevalence in rodents

Out of the 605 examined rodents, the presence of CNM DNA was detected in spleens of 41 individuals. CNM infection rate in Fúgelka was significantly higher than in Bratislava (Fig. 1). The prevalence in rodents captured along the tick collection transects did not differ significantly in any of the two habitats (Table 2). In Bratislava, the number of rodents was lowest along transect B1, where only two uninfected individuals were captured in 2012.

Total CNM prevalence in rodent males and females was 7.5 % and 6.0 %, respectively. The differences between sexes were not significant either for overall prevalence or for prevalence in individual species (Table 3). A comparison of prevalence between years (2012 and 2014; 2013 was not included in the analysis due to the low number of captured specimens) did not reveal any significant differences (Table 4).

**Table 3:** Overall prevalence of CNM in rodents per species, sex and site

		Males		Females		Fisher’s	Total	
Site	Species	% (pos/ex)	95 % CI	% (pos/ex)	95 % CI	exact test	% (pos/ex)	95 % CI
Bratislava	*Apodemus* spp.^a^	3.1 (3/96)	0.0–7.3	2.4 (2/84)	0.0–6.0	1.000	2.8 (5/180)	0.6–5.6
	*Myodes glareolus*	6.2 (4/65)	1.5–12.3	1.9 (1/54)	0.0–5.6	0.375	4.2 (5/119)	0.8–8.4
	Total	4.3 (7/161)	1.2–7.5	2.2 (3/138)	0.0–5.1	0.350	3.3 (10/299)	1.3–5.4
Fúgelka	*Apodemus* spp.^a^	11.2 (11/98)	5.1–18.4	7.5 (6/80)	2.5–13.8	0.452	9.6 (17/178)	5.1–14.0
	*Myodes glareolus*	9.4 (5/53)	1.9–18.9	5.5 (3/55)	0.0–12.7	0.485	7.4 (8/108)	2.8–12.0
	*Microtus* spp.^b^	12.5 (1/8)	0.0–37.5	41.7 (5/12)	16.7–66.7	0.325	30.0 (6/20)	10.0–50.0
	Total	10.7 (17/159)	6.3–15.1	9.5 (14/147)	5.4–14.9	0.850	10.1 (31/306)	6.5–13.7
Total		7.5 (24/320)	4.7–10.6	6.0 (17/285)	3.5–9.1	0.518	6.8 (41/605)	5.0–8.8

*(pos/ex)* number of positive/number of examined, *95 % CI* confidence interval

^a^ *Apodemus* spp. comprise *A. flavicollis* and *A. sylvaticus* (one female from Bratislava, one male from Fúgelka) and one *Micromys minutus* male from Fúgelka

^b^ *Microtus* spp. comprise *Microtus arvalis* and one *Microtus subterraneus* male

**Table 4:** Overall prevalence of CNM in rodents per site in 2012–2014

	2012		2013		2014		Fisher’s exact
Site	% (pos/ex)	95 % CI	% (pos/ex)	95 % CI	% (pos/ex)	95 % CI	Test^a^
Bratislava	4.3 (8/184)	1.6–7.6	16.7 (1/6)	0.0–50.0	0.9 (1/109)	0.0–2.8	0.161
Fúgelka	9.9 (22/222)	6.3–14.0	50.0 (1/2)	0.0–100.0	9.8 (8/82)	3.7–17.1	1.000
Total	7.4 (30/406)	4.9–10.1	25.0 (2/8)	0.0–62.5	4.7 (9/191)	2.1–7.9	0.287

*(pos/ex)* number of positive/number of examined, *95 % CI* confidence interval

^a^only years 2012 and 2014 were compared

Total CNM infection rates were significantly lower in rodents captured in spring (April–June) (2.9 %; 95 % CI: 1.0–4.8 %) than in autumn (September–October) (11.0 %; 95 % CI: 7.6–14.8 %) (Fisher’s exact test, *P* < 0.001). Considering habitat, the seasonal differences were statistically significant in Bratislava, but not in Fúgelka (Fig. 2).

Juveniles and sub-adults represented 5.0 % and 3.9 % of the captured rodents in Bratislava and Fúgelka, respectively. Except for a single infected sub-adult *A. flavicollis* captured at Fúgelka in June 2012, all infected rodents were adults. Dependence of CNM infection on rodent age was not evaluated. Out of the 285 captured rodent females, five (1.7 %) were gravid (two in Bratislava and three in Fúgelka). CNM was detected in the spleen and lungs of a single gravid *Microtus arvalis* captured at Fúgelka in September 2012. Its foetuses were not screened for CNM.

CNM was detected in four out of the six captured species: *Apodemus flavicollis*, *Micromys minutus*, *Myodes glareolus*, and *Microtus arvalis*. Significant interspecific differences in overall CNM infection rates were determined: *Apodemus* spp. 6.1 % (95 % CI: 3.6–8.7 %), *M. glareolus* 5.7 % (95 % CI: 2.6–8.8 %), and *Microtus* spp. 30.0 % (95 % CI: 10.0–50.0 %) (Fisher’s exact test, *P* = 0.003). Considering habitat types, interspecific differences were significant in Fúgelka (Fisher’s exact test, *P* = 0.018) due to the presence of *M. arvalis*. In Bratislava, where only *Apodemus* spp. with a dominance of *A. flavicollis* and *M. glareolus* were captured, no interspecific differences were found (Fisher’s exact test, *P* = 0.526).

Simultaneous effect of habitat, year, season, rodent species and sex on the probability of infection with CNM, analysed by logistic regression, resulted in a significant effect of season and species (Table 5).

**Table 5:** Weight and significance of variables remaining in the best selected model for CNM prevalence in rodents

Variable	B	S.E.	Wald	df	*P*	Exp(B)
Habitat (1)	−0.696	0.395	3.101	1	0.078	0.499
Season (1)	−1.313	0.406	10.438	1	0.001	0.269
Genus			9.057	2	0.011	
Genus (1)	−1.447	0.566	6.534	1	0.011	0.235
Genus (2)	−1.792	0.598	8.979	1	0.003	0.167
Constant	−0.410	0.517	0.628	1	0.428	0.664

Categorical variables codings: Habitat (1), urban/suburban habitat = Bratislava; Season (1), Spring; Genus (1), *Apodemus*, Genus (2), *Myodes*; variables removed by backward method were sex; B, parameter estimate; Wald, Wald statistic = test of significance of the regression coefficient; *P*, significance level

Screening of blood, skin and inner organs (lungs, kidney, liver) of rodents with positive spleens showed the highest infection rate for lungs (100 %) and the lowest for liver (45.9 %) (Table 6). In a *M. glareolus* male captured in Bratislava in September 2012, CNM was detected only in skin, but not in the other examined organs. This specimen was not included as positive in the statistical analyses.

**Table 6:** Detection of CNM DNA in organs of rodents with positive spleens

Species	Spleen	Lungs	Liver	Kidney	Blood	Skin
	pos	pos/ex	pos/ex	pos/ex	pos/ex	pos/ex
*Apodemus flavicollis*	21	21/21	10/18	17/21	13/20	12/21
*Micromys minutus*	1	1/1	1/1	0/1	0/1	0/1
*Myodes glareolus*	13	12/12	6/12	13/13	8/12	8/12
*Microtus arvalis*	6	6/6	0/6	2/6	2/3	3/6
Total	41	40/40 ^a^	17/37 ^a^	32/41	23/36 ^a^	23/40 ^a^
**%**	**100**	**100**	**45.9**	**78.0**	**63.9**	**57.5**

*pos* number of positive, *pos/ex* number of positive/number of examined

^a^the numbers of screened organs are lower than the number of spleens as not all organs were available

### CNM in rodent-attached ticks

In total, 998 rodent-attached ticks were screened: 933 *I. ricinus* (905 larvae, 27 nymphs, one female), 60 *H. concinna* (56 larvae, four females), 4 *Ixodes trianguliceps* (two larvae, two nymphs) and one *Dermacentor reticulatus* larva. CNM was detected only in *I. ricinus* (11 larvae, one nymph) with the prevalence of 1.3 % (95 % CI: 0.6–2.0 %; Table 7). Infection rate in rodent-attached ticks did not differ significantly between the two habitats (Fisher’s exact test, *P* = 0.143), although it was higher in the natural habitat (2.0 %; 95 % CI: 0.7–3.4 %) than in the urban/suburban habitat (0.8 %; 95 % CI: 0.0–1.7 %).

**Table 7:** Prevalence of CNM in rodent-attached *Ixodes ricinus* per site and year

Site	Year	*I. ricinus* pos/ex			Total
		Larvae	Nymphs	Adults	% (pos/ex)
Bratislava	2012	1/213			0.5 (1/213)
	2013	2/30	1/21		5.9 (3/51)
	2014	0/262			0.0 (0/262)
	Total	3/505	1/21		0.8 (4/526)
Fúgelka	2012	3/169	0/1	0/1	1.8 (3/171)
	2013	0/5			0.0 (0/5)
	2014	5/226	0/5		2.2 (5/231)
	Total	8/400	0/6	0/1	2.0 (8/407)
Total		11/905	1/27	0/1	1.3 (12/933)

*pos/ex* number of positive/number of examined

Twenty out of the 41 CNM-positive rodent individuals were infested with ticks, but only six *A. flavicollis* (30.0 %) carried CNM-positive ticks (in total eight larvae and one nymph). The positive ticks were attached to individuals with positive blood and skin (Table 8). Three (21.4 %) out of the 14 examined rodents with CNM-positive spleens and carrying uninfected ticks were positive in skin and blood, five (35.7 %) were positive in blood but negative in skin, and the rest were positive in inner organs only (Table 8). In the majority of cases, CNM-positive ticks co-fed with uninfected ones on the same infected rodent host. All positive ticks were engorged, whereas negative ticks co-feeding with them were unengorged. However, a few CNM-negative engorged larvae were also collected from rodents with positive blood and skin. In addition, three single positive larvae were obtained from CNM-negative rodents: two *M. glareolus* from Bratislava, one *M. arvalis* from Fúgelka, all captured in 2012.

**Table 8:**
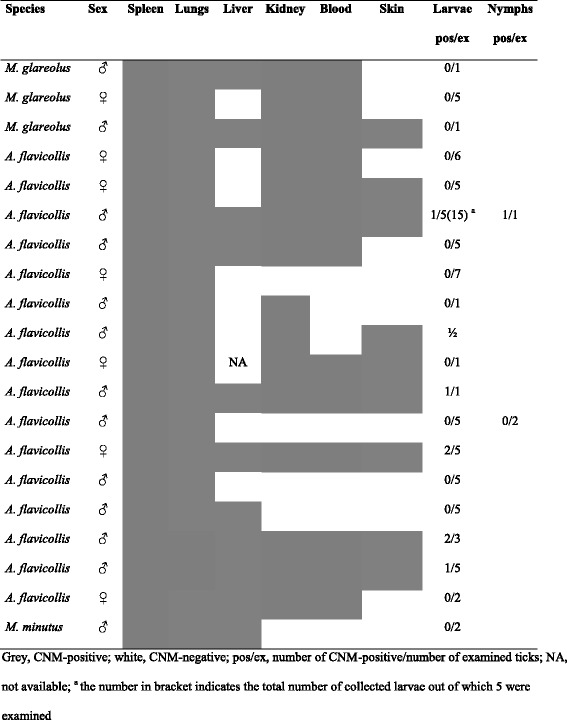
Dissemination of CNM in rodents infested with CNM-positive and CNM-negative ticks

### Molecular analysis of DNA from rodent spleen

A 1024-bp fragment of the CNM *groEL* gene was amplified from three out of six tested rodent spleen DNA (one *A. flavicollis* and two *M. glareolus,* all captured at Fúgelka in 2012). The three DNA sequences were 100 % identical compared to each other and shared 100 % identity with the CNM *groEL* gene amplified from urban hedgehogs in Hungary (KF803997.1), from *I. ricinus* ticks from Poland (KF312363) and from a patient with severe febrile illness in Germany (EU810406). The sequence from *M. glareolus* was submitted to the NCBI database (accession number KR912350).

## Discussion

### Prevalence of CNM in questing ticks

In our study, CNM was detected only in questing *I. ricinus*, the most abundant tick species in Europe [37] as well as in our study sites [36]. *Haemaphysalis concinna* were CNM-negative, which corroborates findings of other studies suggesting that *I. ricinus* is the primary vector of CNM in Europe [4].

Urban parks and forests represent unique habitats where large populations of *I. ricinus* can develop due to the presence of tick-feeding hosts [38]. CNM was confirmed in *I. ricinus* ticks from urban sites throughout Europe [10, 13, 19–22, 34, 35, 39], including Slovakia [18, 32]. In our study, the total CNM prevalence in questing *I. ricinus* was significantly lower in Bratislava than in Fúgelka and local variations within each habitat type were also observed. The CNM infection rate was within the range published from other European sites, although in particular locations considerably higher prevalence was determined [13, 32, 40]. We assume that the observed spatial variations in CNM prevalence were associated with the diversity of the vertebrate host communities and the presence and abundance of competent reservoirs, such as rodents, in this case [20, 23]. This assumption is supported by the 0.1 % CNM prevalence in ticks from transect B1 (the SAS campus), where the lowest rodent density (0.7 individuals/100 trap nights) was determined. In contrast, CNM prevalence in ticks ranged from 1.5 to 3.6 % in locations with denser rodent populations, i.e. 19.8 and 16.1 individuals/100 trap nights in Bratislava forest park and Fúgelka, respectively. However, prevalence data from different studies should be compared with caution due to the sensitivity of the molecular methods used for CNM detection (e.g. conventional PCR versus real-time PCR) and differences in the tested sample sizes.

Unlike other tick-pathogen associations, e.g. *A. phagocytophilum* for which infection rates in adult questing ticks tend to be higher than in nymphs [36], the predominance of CNM infection could not be found in any of the tested tick developmental stages or sexes. Year-to-year and seasonal variation in CNM prevalence in questing ticks, but without clear trends, observed in our study sites was similar to other locations throughout Europe [12, 19, 20, 35]. We assume that the factors affecting temporal changes and interstadial differences in tick-borne pathogen prevalence include global factors (e.g. climate, weather), microclimatic conditions and availability of tick-maintenance and reservoir hosts. The interaction of these factors probably influences the phenology of the vector tick and the transmission paths of tick-borne microorganisms in individual locations and time points, as suggested in [35].

By simultaneously screening *I. ricinus* for CNM and *A. phagocytophilum* we detected only 0.1 % of co-infections with the two bacteria, which is in agreement with results from other European sites [10, 17, 19, 34, 35]. This finding, along with the lower proportion of CNM- and the higher proportion of *A. phagocytophilum*-infected ticks in a site with low rodent and high roe deer density (transect B1) [see above and in 36] support the assumption that CNM and the *A. phagocytophilum* strains transmitted by *I. ricinus* do not share the same reservoir hosts in SW Slovakia.

### Prevalence of CNM in rodents and rodent-attached ticks

The reservoir competence of rodents for CNM was indicated by molecular detections of the bacterial DNA in field-trapped rodents and rodent-attached ticks [12, 13, 17, 24, 25, 31, 41–43] and has been confirmed by a recent xenodiagnostic study [23]. Moreover, the proof for transplacental transmission suggested that CNM is mainly a rodent-associated pathogen [20]. The overall CNM prevalence in rodents from our study area is lower than, e.g. in Eastern Slovakia (8.6–27.5 %) [31], the Netherlands (16.2 %) [12], Germany (14.2–58.5 %) [13, 17, 20] or Sweden (8.8–19 %) [24, 43], but is higher than, e.g. in France (1.8 %) [25], Hungary (3.4 %) [42], or in Switzerland (3.9 %) [23]. But again, the results need to be compared with care since the techniques used to detect the CNM DNA (PCR versus real-time PCR) and the target organs (spleen versus blood) were not the same in the mentioned studies. Nevertheless, the approximately four times higher infection rate in rodents in comparison with questing ticks from our study area supports conclusions of previous studies on the reservoir role of rodents for CNM.

Similarly to questing *I. ricinus*, CNM prevalence in rodents was significantly higher in the non-fragmented forest than in the urban/suburban habitat. This fact could partly be explained by the different spectrum of rodent species, but also may depend on the living conditions and population densities of individual species in different habitat types, as suggested, e.g. by [20]. Among the six rodent species captured, CNM was detected in the most numerous species, *A. flavicollis* and *M. glareolus*, in *M. arvalis* but also in a single *M. minutus* specimen. To the best of our knowledge, this is the first report on the detection of CNM in *M. minutus.*

Temporal fluctuations in rodent populations are known to influence infections with rodent-borne pathogens and disease outbreaks [44]. Significant year-to-year differences in the prevalence of CNM in rodents, determined e.g. in particular sites in Germany [20], were not confirmed in our study sites. On the other hand, increases in CNM infection rates in rodents captured in autumn were also reported in Germany and Southern Sweden [13, 43]. In contrast to a recent study from Germany, where a higher proportion of infected rodent males than females was determined [20], we did not find any sex-related difference for CNM. With regards to rodent age, our findings were in line with a study from Sweden [43], where no infection was detected in juveniles and the infection rate in sub-adults was lower than in adults. These results were in contrast with recent detections of CNM in rodent foetuses and neonates from Germany [20]. Obviously long-term field investigations along with experimental studies are necessary to understand temporal fluctuations in CNM prevalence and transmission paths of the bacterium in rodent populations.

Our results confirmed, in part, previous findings on CNM dissemination in organs of field trapped rodents [12, 13] and suggested that, in addition to spleen and kidney, lungs could be added to the list of organs suitable to screen wild rodent populations for CNM prevalence. In contrast to findings on the low infection rates in skin [13], but in line with only a two times higher prevalence in the spleen compared with skin, reported in another study [42], we detected CNM in 57.5 % of ear biopsies of rodents with positive spleens. Moreover, we detected CNM exclusively in the skin of one *M. glareolus*. No attached ticks were collected from this specimen, but we assumed that this local infection could be due to infestation with CNM-positive tick(s) that detached shortly before the rodent was captured and before dissemination of the bacterium could take place. In summary, results on detections of CNM in organs of rodent specimens originating from a wild population may display various stages of infection, but also variations in the course of infection in different species.

In our study, the CNM prevalence in rodent-attached ticks was similar to the prevalence in questing ticks, but it was approximately five times lower than in rodents. Our results contradict the 2.6 % prevalence in rodent-attached ticks determined along with a 3.9 % infection rate in rodents in Switzerland [23], but seem to correspond with results of a recent study from Germany [20]. We detected CNM also in a few *I. ricinus* larvae feeding on uninfected rodents. As transovarial transmission of the bacterium has not been confirmed in ticks [4, 23], we assumed that the larvae acquired infection by pre-feeding on an infected host. We found that the majority of CNM-positive ticks attached to infected rodents were engorged. However, a few negative engorged ticks were also gathered from rodents with positive blood. Thus we assume that, similarly to other tick-borne pathogens [45], the course of infection of ticks and rodents with CNM depends on still unknown physiological and molecular interactions on the tick-host-pathogen interface.

For a number of CNM genotypes from *I. ricinus* ticks and rodents from Europe, identity with the CNM genotype that caused disease in humans in Germany has been confirmed [12, 22, 25, 46, 47]. The sequences obtained from infected rodent spleens in our study showed the highest identity with the *groEL* gene sequences from infected human blood from Germany [47], *I. ricinus* ticks from Poland [22] and urban hedgehogs from Hungary [26]. Thus our results confirm the presence of a human pathogenic CNM genotype in South-Western Slovakia and indicate that there is a risk for humans to contract CNM infection.

## Conclusions

The present study showed that CNM was present in questing and rodent-attached *Ixodes ricinus* ticks and rodents in urban, suburban and natural habitats of South-Western Slovakia. Spatial and temporal variations in CNM prevalence in ticks and rodents were observed, depending on habitat type, location and season. Interspecific differences in CNM prevalence in rodents with the highest infection rate in *Microtus arvalis* were revealed. Detections of CNM in rodents suggested their reservoir role for the bacterium and their epidemiological significance in the maintenance of CNM in natural foci of Slovakia. The identity of CNM gene sequences from infected rodent spleen with gene sequences from infected human blood was confirmed. Although no human CNM infections have been reported in Slovakia, our results confirmed the presence of a human pathogenic CNM genotype in South-Western Slovakia and indicated a potential risk of contracting infections in humans bitten by ticks. However, further investigations of the tick-host-pathogen interactions are necessary to understand CNM transmission pathways in natural foci and the relevance of CNM to public health.
